# 

**Published:** 1900-02-24

**Authors:** 


### NOTICE TO CORRESPONDENTS.

All MS., Letters, Books for Review, and other matters intended for the Editor should be addressed The Editor, "The Hospital," The Hospital Building, 28 & 29, Southampton Street, Strand, W.C.

The Editor cannot undertake to return rejected MS., even when accompanied by stamped directed envelope.

**Notice to Advertisers and Subscribers.**

All Advertisements, Orders for Copies of The Hospital, and Business Communications should be addressed to **THE MANAGER (Not to the Editor)**, The Hospital, The Hospital Building, 28 & 29, Southampton Street, Strand, London, W.C.

The Cost of Subscription, post free, is as follows:—Per week, 2½d. per quarter, 2s. 8d.; per half-year, 5s. 3d.; per annum, 10s. 6d. Foreign:—Per annum, 15s. 2d., payable, in all cases, in advance.

**NOTICE.—Vol. XXVI. of "The Hospital" (April, 1899, to September, 1899), in handsome cloth binding, is now on sale at the Office of this Journal, price 7s. 6d. Cases for binding the Numbers contained in this Volume 1s. 6d. Index to Vol. XXVI., 2½d., post free.**

**The Monthly Part for March will be ready on March 1st. Price 6d., by post 8d.**

### BOOKS RECEIVED.

Scientific Press.

"The Nursing Profession: How and Where to Train." Edited by Sir Henry Burdett, K.C.B. Second year.

Swan Sonnenschein and Co.

"Consumption and Chronic Disease." By Emmet Densmore, M.D.

The "Municipal Journal" Office.
"The Municipal Year Book of the United Kingdom" for 1900.

"Edinburgh Medical Journal."

Reprints of Papers on "Strumous Ophthalmia" and "Purulent Ophthalmia." By A. Maitland Ramsay, M.D.

H. K. Lewis.
"Mentally Deficient Children: Their Treatment and Training." By G. E. Shuttleworth, B.A., M.D. Second Edition.

### The Student of Medicine.

### APPOINTMENTS.

M. Aungier, M.B., Ch.B.Vict., appointed Senior House Surgeon to the Bootle Hospital, Liverpool. J. H. Arbuckle, M.D.Glasg., D.P.H.Camb., appointed Medical Officer of Health for Kilmarnock. S. E. Baxter, M.R.C.S., L.R.C.P., appointed Medical Officer and Public Vaccinator for the Wollaston District of the Wellingborough Union. E. J. A. Chatelier, M.B., C.M.Edin., appointed Medical Superintendent of the City Hospital, Birmingham. S. C. Harris, L.F.P.P Glasg., L.M., L.S.A., appointed Medical Officer of the St. Mary's District of the Ely Union. G. A. Hutchinson, M.R.C.S., L.R.C.P., appointed Assistant Medical Officer to the Glamorganshire County Asylum, Bridgend. R. Riddell, M.B., C.M.Edin., appointed Medical Officer for the Workhouse and the Sandbach District of the Congleton Union. A. H. Robinson, M.D.Durh., M.R.C.S, D.P.H.Camb., appointed Medical Superintendent of the Islington Infirmary, Highgate Hill, N. C. J. Staunton-Cahill, L.R.C.P., L.R.C.S.I., appointed Junior House Surgeon to the Bootle Hospital, Liverpool.

### Scraps and Gleanings.

During the past year the earnings of the private nursing department of the Hertford General Infirmary amounted to £426 odd; the number of cases attended by this department was 76.

The committee of the Dewsbury and District Infirmary reported at the annual meeting a very satisfactory condition of affairs; after maintaining the building, &c., there was a balance of something like £100 to the good.

The committee of the Denbighshire Infirmary have offered the War Office six beds for the use of sick and wounded soldiers and sailors on certain terms: (1) a charge of 2s. per day for each bed, whether used or not; (2) no infectious case would be admitted.

Of the total revenue of the Glasgow Eye Infirmary for last year, viz., £4,346, the sum of £2,458 was contributed by public works. The total ordinary expenditure for the year was £4,210 odd, a balance of £135 odd being carried to extraordinary account. The reserve fund during the year had benefited by £1,051.

A fund has been started for the purpose of effecting some improvements and alterations at the Peterborough Infirmary, but no action is to be taken on the part of the committee to extend this fund until a definite scheme has been agreed upon. The committee have in view a scheme for the extension of the infirmary at a probable cost of £1,200 or £1,500.

The Perth City and County Infirmary was described at the recent annual meeting as slowly bleeding to death in regard to its financial position. Consequent upon a suggestion made at the meeting the Rev. A. Fleming, who is senior minister of Perth, will call a meeting of ministers for the purpose of considering the question of a Hospital Sunday being held every year on behalf of the Perth Institution.

The seventy-second annual report of the Dover Hospital shows that 350 in-patients were received during 1899, the total cases treated being 4,775. The receipts included £265 from the Hospital Saturday Fund (of which £25 was apportioned to the children's ward), and amounted altogether to £1,713, against an expenditure of £1,735. The deficit of £22 would be taken from the £120 legacies received, so that the year would be started square. The medical officer, Dr. Parsons, and Dr. Osborn, who for 26 years had acted as honorary surgeon, both retire from their respective posts for private reasons.

The Royal Cornwall Infirmary have had some tough legal fighting in respect to a legacy of £800 left them by the late Mr. P. S. Glubb, of Falmouth, who attached to the legacy certain conditions which the Master in Chancery considered had not been fulfilled. An appeal from his decision was made by the infirmary, but the original decision was upheld by Mr. Justice North. The infirmary committee, however, feeling that this decision was opposed to the will of the testator and so unfair to the subscribers, carried the case to the Court of Appeal and the decision has been reversed, so that the infirmary will get now £600 or £650, in addition to £100 already received, as well as £1,000 raised by private subscription to meet one of the conditions attached to the legacy.

The War Office have accepted from Mr. Mariani, of Paris, a consignment of 24 dozen Mariani wine. This, together with a previous contribution of 18 dozen to the "Maine," makes a total of 503 bottles which he has given for the benefit of our sick and wounded.

An excess of expenditure over income to the amount of £44 odd was reported at the annual meeting of the governors of Forres Leanchoil Hospital. The number of patients treated during the year shows an increase of 24, which makes the largest number in any one year since the hospital was opened. The management decided to set aside two beds for convalescent soldiers and sailors from South Africa.

The Secretary of the Tunbridge Wells Homœopathic Hospital and Dispensary, at the recent annual meeting, stated that during the year they had received legacies amounting to £637. The subscriptions, however, showed a decrease of £20, and the donations of £66. Of the 101 patients treated during the year not a single one had died. The Chairman suggested that the clergy of the town should fairly divide among the various hospitals the Hospital Sunday collections, instead of sending only what was specially marked for that hospital.

A very good year's work was reported at the annual meeting of the governors of the Stroud General Hospital. The balance-sheet showed that there had been a decrease in expenditure, principally in the items of "medicine, dressings, &c.," which had been reported last year as being "capable of reduction." During the year the hospital received over £800 in legacies. The committee are considering the question of providing increased storage for water, as during the dry season they had been put on short commons. A scheme for softening the water is also being considered.

At the recent annual meeting of the supporters of the Frome Cottage Hospital the Rev. W. A. Duckworth submitted a resolution, for, if necessary, reserving a ward for the nursing, free of cost, of any wounded Somerset men invalided home from the war. This resolution was duly passed. A resolution was also proposed by a clergyman of the district to the effect that subscribers to the church offertory should receive tickets of admission for patients recommended by them. As, however, the chairman stated, this proposal was a distinct alteration of the rules, it was decided to withdraw it until the new hospital is opened and then give notice of motion.

In a series of articles in the *Manchester Guardian* on the Manchester Royal Infirmary Dr. D. J. Leech, the senior physician, who has long identified himself with the interests of the institution, brings out some very interesting facts. He states that during the past 20 years the number of accidents brought to the infirmary has nearly doubled; that the number of out-patients during the same period has more than doubled; whilst, owing to the existing beds in the hospital (287) having been worked continuously to their utmost capacity, numbers of in-patients have had to be turned away or discharged too soon, and the requirements of this department demand that the number of available beds should be increased to 500.

Demy 8vo., with two Plates, cloth gilt, 12s. 6d. net.

### MEDICAL HISTORY FROM THE EARLIEST TIMES.

By E. T. WITHINGTON, M.A., M.B.Oxon.

"One of the best attempts that has yet been made in the English language to present the reader with a concise epitome of the history of medicine, and as such we very cordially commend it."—*Glasgow Medical Journal.*

London: THE SCIENTIFIC PRESS, LTD., 28 & 29, SOUTHAMPTON STREET, STRAND, W.C.

**NOW READY,**

A REPRINT OF

### A NURSING HANDBOOK

TOGETHER WITH S. MAW, SON, AND THOMPSON'S

**COMPLETE CATALOGUE OF NURSING REQUISITES.**

The above work is now republished at a cost of 1s. per copy. Messrs. S. M., S., and T. have been compelled to make this charge on account of the extreme popularity of the work and the great expense incurred in its production.

***Post Free on receipt of 1s.***

### S. MAW, SON, and THOMPSON,

7 to 12, ALDERSGATE STREET, LONDON, E.C.

